# From association to mechanism in complex disease genetics: the role of the 3D genome

**DOI:** 10.1186/s13075-018-1721-x

**Published:** 2018-09-29

**Authors:** Yao Fu, Kandice L Tessneer, Chuang Li, Patrick M Gaffney

**Affiliations:** 1 Division of Genomics and Data Sciences, Arthritis and Clinical Immunology Research Program, Oklahoma Medical Research Foundation, 825 Northeast 13th Street, Oklahoma City, OK 73104 USA; 2 School of Electrical and Computer Engineering, University of Oklahoma, Devan Energy Hall 150, 110 West Boyd Street, Norman, OK 73019 USA

**Keywords:** Autoimmune disease, 3D genome, Chromatin conformation, Complex genetic disease, DNA looping, Functional genomics, GWAS, Enhancer, Promoter

## Abstract

Genome-wide association studies (GWAS) and fine mapping studies in autoimmune diseases have identified thousands of genetic variants, the majority of which are located in non-protein-coding enhancer regions. Enhancers function within the context of the three-dimensional (3D) genome to form long-range DNA looping events with target gene promoters that spatially and temporally regulate gene expression. Investigating the functional significance of GWAS variants in the context of the 3D genome is essential for mechanistic understanding of these variants and how they influence disease pathology by altering DNA looping between enhancers and the target gene promoters they regulate. In this review, we discuss the functional complexity of the 3D genome and the technological approaches used to characterize DNA looping events. We then highlight examples from the literature that illustrate how functional mapping of the 3D genome can assist in defining mechanisms that influence pathogenic gene expression. We conclude by highlighting future advances necessary to fully integrate 3D genome analyses into the functional workup of GWAS variants in the continuing effort to improve the health of patients with autoimmune diseases.

## Background

Genome-wide association studies (GWAS) have significantly advanced the identification of variants associated with complex genetic diseases, including autoimmune diseases [1, 2]. GWAS leverages the phenomenon of linkage disequilibrium—the tendency for common variants to be inherited in correlated haplotype blocks—to identify statistical associations between genetic diseases and haplotypes of single nucleotide polymorphisms (SNPs) [3]. Statistical associations, while powerful for locus discovery, cannot distinguish risk-driving variants within a haplotype block that are responsible for the genetic association from non-risk neutral variants. Large-scale GWAS have shown that the vast majority (~ 80–90%) of GWAS variants are located in regions of genomic DNA that do not code for protein sequences [4, 5]. These variants are thought to exert their influence on disease risk by modulating gene expression, which can vary based on cell type and cell state. Compared to genetic variants in protein coding sequences for which the impact of an amino acid change on protein function can be reasonably predicted, the function of non-coding DNA variants must be empirically determined through experimentation, hindering translation of GWAS data into clinically meaningful information.

To demystify the function of non-coding DNA in chromatin regulation and gene expression, several large-scale collaborative efforts (Encyclopedia of DNA Elements (ENCODE) Project, National Institutes of Health (NIH) Roadmap Epigenomics Mapping Consortium, and International Human Epigenome Consortium (IHEC)) have successfully mapped the locations of regulatory sequences that bind over 400 transcription factors and histone post-translational modifications that mark enhancers, promoters, repressors, and insulator regions in a large variety of cell lines and primary cells [6–8]. Collectively, these studies provided a detailed “parts list” of non-coding DNA elements, suggesting that over 80% of what was once referred to as “junk DNA” may have a role in gene regulation [6]. With this “parts list” and their precise genomic locations, it is now possible to develop and test functional hypotheses about how variants associated with complex genetic diseases potentially alter the function of enhancers to influence the expression of target genes.

**Fig. 1:**
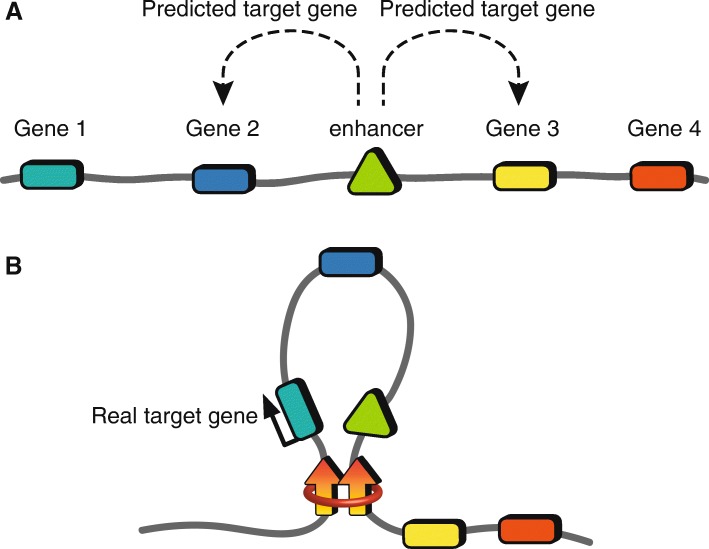
Predicting enhancer–promoter interactions using linear proximity versus 3D proximity. **a** Traditional modeling of enhancer function in the context of a linear genome where an enhancer (*green triangle*) is predicted to modulate the function of the promoter in closest linear proximity (gene 2 (*blue rectangle*) or gene 3 (*yellow rectangle*)). **b** Modeling in the context of the 3D genome where an enhancer (*green triangle*) often regulates distant gene expression through long-range DNA looping to the gene promoter (gene 1 (*green rectangle*)). Due to spatial proximity, the enhancer “skips” gene 2 (*blue rectangle*). Enhancer function is restricted within the insulated loop structure formed by a CTCF-CTCF (*arrows*)–cohesion (*red ring*) complex, and therefore cannot activate gene 3 (*yellow rectangle*) or gene 4 (*red rectangle*) despite close linear proximity

Enhancer elements are short DNA sequences (~ 50–1500 bp) that bind transcription factors leading to the expression of a gene [9]. It is estimated that the human genome has nearly one million enhancer sequences scattered throughout all 23 pairs of chromosomes, a number that far exceeds the estimated 20,000 genes in the human genome [9, 10]. Moreover, approximately 60% of autoimmune disease GWAS variants reside in enhancer elements, suggesting that much of autoimmune disease risk is concentrated on modulating gene expression [5]. Enhancers influence gene expression by delivering their payload of transcription factors to the gene promoter most often located on the same chromosome, but at varying distances, through a process of DNA looping [11]. The mechanisms that govern DNA looping and the technologies to measure them are a burgeoning area of research and have been the subject of many detailed reviews [11–15]. Knowledge of DNA looping mechanisms is important because it reveals how specific enhancer–promoter interactions occur and are modulated in response to specific cellular contexts. Traditionally, it has been naively assumed that the gene promoter closest to an enhancer is the target promoter that is regulated by that enhancer (Fig. 1a); however, we now know that enhancers likely engage multiple distant promoters within an enhancer’s “regulatory network”—defined as all physical interactions between a given enhancer and gene promoters in the region (Fig. 1b) [16, 17]. Furthermore, formation of enhancer regulatory networks is likely cell type-specific and influenced significantly by autoimmune disease-associated SNPs enriched in the enhancer region [17].

In this review, we discuss the functional complexity of the 3D genome, contrast the various aspects of how the 3D genome is measured, and provide specific examples of how knowledge of the 3D genome has helped decipher GWAS results. We conclude by highlighting future advancements needed to generalize 3D genome data into the routine analysis of GWAS-associated risk variants.

## Main Text

### Functional complexity of the 3D genome

**Fig. 2:**
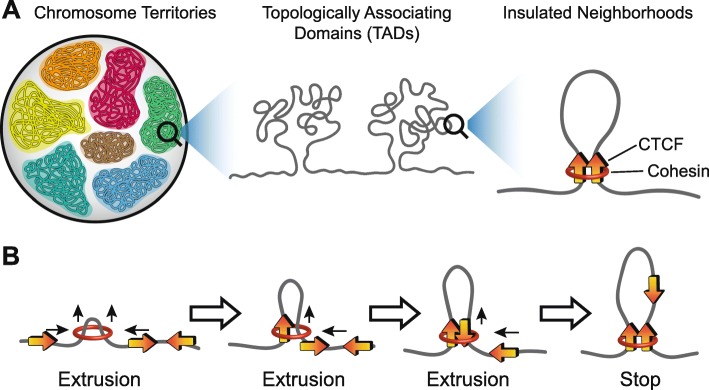
3D genome organization. **a** Each chromosome tends to occupy a particular region in the nucleus, defined as chromosome territories. Within a chromosome, there are regions with relatively high interaction frequencies, defined as topologically associating domains (TADs), and regions with relatively low interaction frequencies called TAD boundaries. Nested within each TAD are several sub-TAD domains, such as insulated neighborhoods, defined as DNA loops formed by CTCF homodimer (*orange arrows*), co-bound with cohesin (*red ring*), and containing at least one gene. **b** Extrusion/sliding model for TAD and sub-TAD loop formation: cohesin ring (*red ring*) facilitates the “sliding” of DNA through the ring structure to form a small loop. When bound CTCF (*orange arrow*) encounters cohesin, the DNA stops sliding on that side. The opposing side continues to slide through until a convergently oriented CTCF anchor motif is recognized and the insulator CTCF-CTCF–cohesin complex forms. Loops are less likely to form if two CTCF binding motifs are of tandem or divergent orientation

The human genome is organized into complex layers of intricate folds and loops that allow for proper gene expression regulation while fitting roughly three meters of histone-wrapped DNA into an interphase nucleus averaging 6 μm in diameter [11, 14, 15]. Each of the 23 homologous chromosomes are organized into specific regions of the nucleus, called chromosome territories, that restrict interactions between different chromosomes (Fig. 2a) [18, 19]. Each chromosome undergoes additional organization into active “A” and inactive “B” compartments [20–23]. B compartments contain densely packed regions of DNA, called heterochromatin, that are enriched with histone marks of inactivity [20]. A compartments of DNA are typically areas of open chromatin. A/B compartments are further organized to create thousands of megabase-sized sub-regions, called topologically associating domains (TADs), that promote chromatin interactions within the TAD and restrict interactions outside the TAD (Fig. 2a) [18, 20–24]. TAD boundaries are enriched with CCCTC-binding factors (CTCF) and cohesin proteins which facilitate loop formation through a process of loop extrusion (Fig. 2b) [13, 25, 26]. During loop extrusion, cohesin binds to and facilitates the “sliding” of DNA through the cohesin ring structure. The “sliding” on one side of the small loop tends to stop when a CTCF-bound sequence encounters the cohesin. The other side of the loop continues to “slide” and “grow” until another CTCF-bound sequence with convergent orientation reaches the cohesin. The two CTCF proteins homodimerize and create a stabilizing complex with cohesin [25]. The unknotted loop of DNA “extruding” from the newly established CTCF-CTCF–cohesin complex forms the TAD [25]. TADs are largely evolutionarily conserved and maintained during cellular differentiation and embryonic development [18, 27, 28]. In contrast, CTCF-CTCF–cohesion bound regions known as “insulated neighborhoods” organize dynamic enhancer–promoter interactions during cellular differentiation or in response to stimuli (Fig. 2a) [14, 15, 29, 30]. Typically, more than one dynamic insulated neighborhood is nested within a larger evolutionarily conserved TAD.

Characterizing the complex layers of organization that occur within the 3D genome and the regulatory mechanisms that dictate them have provided a framework from which current explorations of gene expression regulation are often based. Approximately 90% of identified enhancer–promoter loops occur within the CTCF-CTCF–cohesin boundaries of TADs and insulated neighborhoods [31]. As reported in several types of cancer, disrupting these boundaries can alter gene expression by relieving restrictions and allowing new loops to form between what was once an insulated enhancer and genes outside of the original loop [32]. What’s more, enhancer–promoter loops function not in isolation, but as regulatory networks where one enhancer has the potential to influence multiple genes and one gene can be influenced by multiple enhancers [16]. Given that a large majority of GWAS variants are located within enhancers that likely modulate distant, as well as neighboring, gene function, establishing detailed maps of enhancer–promoter loops and regulatory networks have the potential to more accurately predict the functional mechanisms influenced by causal GWAS variants and provide translational insights into how such alterations influence disease pathogenesis [5].

### Investigating the 3D genome

**Fig. 3:**
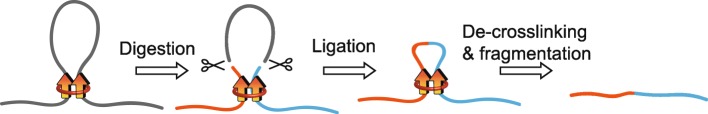
Proximity ligation. Chromatin are crosslinked to preserve interactions between proximal regions of DNA and associated proteins. Crosslinked chromatin are digested using restriction enzymes (*scissors*) to create two short DNA fragments complexed with associated proteins. “Sticky ends” of the two DNA fragments originally in close 3D proximity are then ligated using DNA ligase to create a chimeric strand of DNA. After de-crosslinking, the chimeric DNA can be used in downstream applications to identify and characterize loop formation

The majority of techniques used to investigate the 3D genome are derivatives of the original chromatin conformation capture (3C) method, which uses a process called proximity ligation to capture interactions between two sequences of DNA that are in 3D proximity but are separated by linear distance (Fig. 3) [33, 34]. To capture a long-distance interaction, cells are first crosslinked, or fixed, to preserve interactions between the two regions of DNA and associated proteins, and then the entire genome is digested into small pieces using restriction enzymes. Because crosslinking keeps the interacting regions of DNA and associated proteins in close proximity after digestion, the remaining regions of DNA can be enzymatically ligated together to make a chimeric strand of DNA that, once de-crosslinked, can be used in downstream 3D chromatin applications. Building upon this basic methodologic framework, variations have been developed to facilitate both targeted and genome-wide 3D chromatin exploration.

Targeted hypothesis-driven methodologies are used to analyze looping events for one or more selected targets (Table 1). Original 3C uses a unique set of primers and quantitative PCR to measure the frequency of interactions captured by proximity ligation [35]. Despite low throughput, 3C remains one of the most commonly used methods because it is cost-effective, easily adaptable to PCR-capable laboratories, quantitative, and the unique primers define a specific region of interest, thus providing relatively high resolution of the interacting regions (~ 250 bp to 4 kb) [34, 35]. Typically, 3C is used to confirm suspected looping events and quantitatively measure changes in looping patterns caused by allelic variation in enhancer SNPs. For example, 3C was used to solve the long-time mystery of how paternal imprinting at the insulin-like growth factor 2 (*IGF2*) and *H19* gene loci alters expression of the non-coding RNA, *H19* [36]. 3C revealed an enhancer region that forms either an enhancer–promoter loop with the *H19* gene promoter on the maternal allele or with the *IGF2* gene promoter on the paternal allele. Further functional analyses revealed that a control region upstream of *H19* is methylated on the imprinted paternal allele which blocks looping to the *H19* promoter, thus silencing *H19* expression and activating *IGF2* expression [37, 38]. More recently, 3C has been used to demonstrate how SNPs in loop boundaries and anchors can significantly alter gene expression. In isocitrate dehydrogenase (IDH) mutant gliomas, 3C revealed that a gain-of-function mutation caused hypermethylation at the CTCF binding site defining an insulated neighborhood containing the oncogene, platelet-derived growth factor receptor alpha (*PDGFRA*) [39]. Hypermethylation disrupted formation of the insulated neighborhood, allowing a constitutive enhancer outside of the loop to promote oncogenic expression of *PDGFRA* [39].

**Table 1:** Advantages and disadvantages of current 3D genome technologies

Technique		Assay name/description	Target size	Assay platform	Cell input	Advantages	Disadvantages	References
Targeted								
3C		Chromosome conformation capture	One target	Quantitative PCR	> 100 M	• Quantitative measurement of long-range interactions between two targeted loci • No sequencing required	• Low throughput • Large amount of input cells	[34, 35]
4C		Circular chromosome conformation capture or chromosome conformation capture-on-chip	Multiple targets	Microarray	> 100 M	• Identification of multiple DNA regions that interact with a target locus • Modified protocol: 4C-seq	• Relatively low throughput • Large amount of input cells	[40, 63]
5C		Chromosome conformation carbon copy	Multiple targets	Microarray or sequencing	> 100 M	• Multiplexed conformation capture • Higher efficiency and lower background compared to 3C	• Not all sites are compatible to 5C primer design • 5C cannot detect contacts larger than a few megabases	[41]
Capture-C		3C with specific oligonucleotides capture	Multiple targets	Sequencing	10-20 M	• Unbiased capture of all regions interacting with a specific target sequence • Reduced background signal compared to Hi-C • More informative contacts • Modified protocol: Capture Hi-C	• Interaction detection depends on the design of the target “bait”	[42]
Genome-wide								
Non-protein-mediated	Hi-C	Chromosome conformation capture by high-throughput sequencing	All interactions	Sequencing	20-25 M	• High throughput • Improved efficiency • First genome-wide assay • Modified protocol: in situ Hi-C; single cell Hi-C	• High background due to random ligations • Requires deep sequencing • Relatively low resolution	[21, 44–48]
Protein-mediated	ChIA-PET	Chromatin interaction analysis by paired-end tag sequencing	All interactions	Sequencing	> 100 M	• Identify specific protein-mediated DNA loop structures • Reduced background noise in sequencing data	• Long processing time (> 6 days) • Requires high efficiency ChIP-grade antibodies	[49]
	HiChIP/ PLAC-seq	In situ Hi-C with protein-centric ChIP/proximity ligation-assisted ChIP-seq	All interactions	Sequencing	1-10 M	• Faster protocol (2 days) • Higher efficiency than ChIA-PET • Less sequencing	• Requires high efficiency ChIP-grade antibodies	[50–52]

Innovative modifications to traditional 3C, including methodologies such as 4C, 5C, and Capture-C (Table 1), have coupled 3C with microarray or high-throughput next generation sequencing (NGS) technologies to improve throughput with only minor reductions in resolution, but at the expense of quantitative capabilities [40–42]. Improved throughput has allowed for larger-scale targeted studies, like the Promoter Capture-C study by Hughes et al. that comparatively mapped the interactions between 6000 promoters and their regulatory elements in mouse embryonic stem cells and mature erythroid cells [42]. This study not only demonstrated the complexity of enhancer–promoter networks and that the promoter of a specific gene can be regulated by interactions with multiple regulatory elements, but also provided strong evidence that disease-associated risk variants are enriched in gene regulatory elements such as enhancers. Currently, most targeted methodologies still require over 100 million cells to get chromatin quantities necessary to obtain meaningful results, thus restricting use to immortalized cell lines [15]. Given the cell type- and context-specific nature of chromatin dynamics, restricted use of 3C-based technologies to immortalized cell lines has hindered functional characterization of GWAS variants in more relevant primary cell models.

To capture 3D chromatin interactions on a genome-wide scale, proximity ligation was coupled with NGS to create an innovative method called Hi-C (Table 1) [21]. Following proximity ligation, chimeric strands are sequenced and aligned to a reference genome to identify where the two interacting regions were originally located within the linear DNA sequence, thereby identifying the anchor points where chromatin organizing proteins form a DNA loop. Early investigations using Hi-C revealed, for the first-time, chromatin substructures, i.e., TADs, within the context of previously characterized chromosome territories [18]. Subsequent advances in Hi-C methodology have improved resolution from > 100 kb in 2012 to ~ 5–10 kb in 2017, allowing for the generation of 3D genome maps that are now widely used to predict enhancer–promotor interactions occurring within a population of cells at a fixed time [28, 43–45]. Javierre et al. used promoter capture Hi-C (PCHi-C) to map the interacting regions of 31,253 promoters in 17 human primary blood cell types [46]. Not only did this study successfully use primary human cells to perform PCHi-C, but also successfully demonstrated that active enhancers significantly and quantitatively contribute to cell type-specific promoter activity and subsequent gene expression [46].

Single cell Hi-C was first reported in 2013 to explore the cell-to-cell variability of chromatin structures using a single copy X-chromosome model in isolated mouse nuclei [47]. More recently, the use of nucleic acid barcodes to index single cell nuclei eliminated the need to isolate individual nuclei for Hi-C, thus providing a more streamlined approach [48]. As single cell technologies improve along with analytical methods, we anticipate rapid adoption of single cell 3D genome approaches for many experimental designs.

Protein-mediated genome-wide methodologies (Table 1) include additional steps to isolate regions of interacting DNA based on the architectural proteins that influence those interactions, such as loop boundary markers (CTCF, cohesin, etc.), epigenetic markers of enhancers (acetylation of histone H3 on lysine 27 (H3K27ac)), or transcription factors [15, 33, 49–51]. Targeting specific proteins involved in chromatin organization reduces the background signal and the required sequencing depth—number of sequencing reads—necessary to achieve meaningful semi-quantitative results. Furthermore, these improvements have significantly reduced the number of cells required, making it possible to now study DNA looping in primary cells. Recently, Mumbach et al. [52] reported using between 0.5 and one million cells to identify H3K27ac (a histone modification of active enhancers) looping profiles on naïve T cells, T-helper, and Th17 cells isolated from a primary T-cell population using HiChIP technology. The study demonstrated unique and differentially active enhancer loop clusters that corresponded with altered gene expression in each cell type [52], thus supporting current models suggesting different cell types and cell states adopt modified regulatory networks with specific enhancer–promoter loops to drive unique gene expression profiles.

3D genome-wide exploration generates tremendous amounts of sequencing data that require advanced algorithms and pipelines for processing, visualizing, and interpreting the functional significance of these 3D features. Fortunately, several robust software packages are publicly available and more are in development. Each algorithm uses different alignment strategies and filtering criteria to generate heatmaps based on interaction frequencies [43, 53] or looping diagrams that map protein-mediated DNA looping events in the context of linear chromatin [54]. Improvements to capture technologies that select for specific chromatin characteristics, such as histone marks or protein factors, and sequencing technologies that allow for sample barcoding and deeper sequencing continue to improve throughput and reduce background. Simultaneous improvements to the analysis pipelines that define the 3D genome continue to improve the base-pair resolution and quantitative capabilities of 3D technologies, allowing investigations of how disease-associated SNPs alter gene expression through modified 3D genome structures.

### Application of 3C technologies to uncover new insights from GWAS in autoimmune disease

**Fig. 4:**
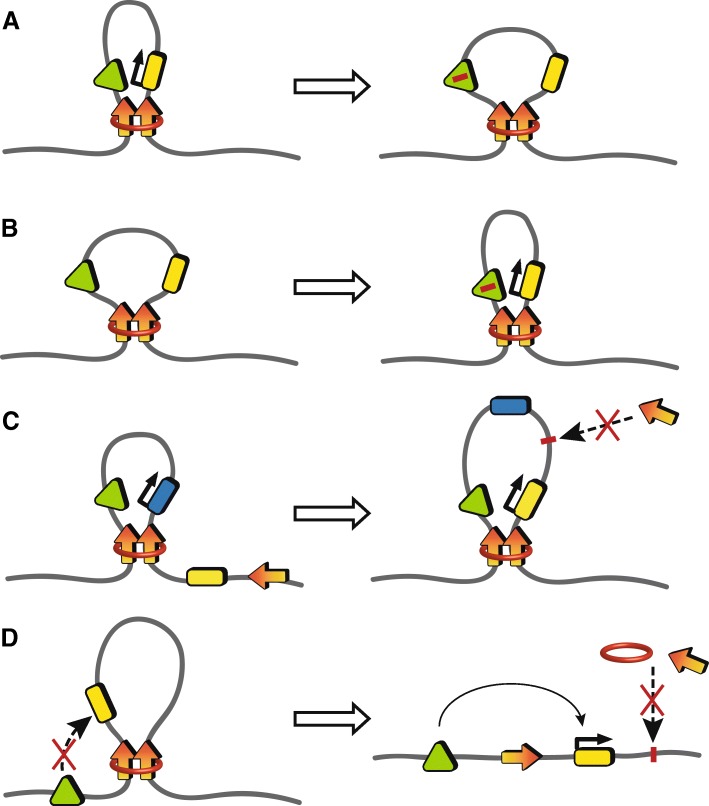
Altering the 3D genome architecture disrupts gene expression regulation. **a**, **b** An enhancer (*green triangle*) can modulate gene expression by interacting with and delivering transcription factors to its target gene promoter (*yellow rectangle*) through long-range enhancer–promoter interactions. A causal mutation (*red bar*) in the enhancer can alter gene expression by modulating the frequency of this interaction. Impairing the frequency of the long-range interaction reduces delivery of transcription factors to the promoter, thus hindering gene expression (**a**). Enhancing interactions between the enhancer and promoter facilitates gene expression (**b**). **c**, **d** Insulated neighborhoods can regulate gene expression by restricting interactions between active enhancers (*green triangle*) and target gene promoters (*blue rectangle*) within an insulated loop boundary. Causal mutations (*red bar*) that disrupt CTCF anchor motifs can modify (**c**) or disrupt (**d**) existing loops, allowing the once-restricted enhancer (*green triangle*) to now interact with gene promoters (*yellow rectangle*) outside of the original insulated neighborhood

Autoimmune diseases, like most complex genetic diseases, result from the collective influence of multiple genetic variants on gene expression and responses to potentially damaging environmental conditions [2]. Investigating the functional significance of GWAS variants in the context of the 3D genome is essential for mechanistic understanding of how these variants, most of which are enriched in largely uncharacterized enhancer regions, influence disease pathology by reducing or enhancing interactions between enhancers and promoters within the enhancer regulatory network (Fig. 4a, b). For example, GWAS and fine-mapping revealed several autoimmune disease risk variants in the chromosome 6q23 locus, including a tandem pair of systemic lupus erythematosus (SLE)-associated polymorphisms, rs148314165 (−T) and rs200820567 (T > A) (referred to as the TT > A variants), located in an ENCODE-identified putative enhancer region located ~ 42 kb downstream of the tumor necrosis factor alpha-induced protein 3 (*TNFAIP3*) gene promoter [55, 56]. *TNFAIP3* is a critical negative regulator of pro-inflammatory nuclear factor kappa B (NF-κB) signaling implicated in many autoimmune diseases, and therefore a suspected target gene of the identified enhancer [55–57]. Functional studies using the quantitative-PCR-based 3C method determined that this enhancer facilitated *TNFAIP3* gene expression by bringing transcription factors, including NF-κB, to the *TNFAIP3* promoter region via long-range enhancer–promoter interactions [55]. Importantly, the presence of the risk allele (−A/−A) in the enhancer was shown to significantly disrupt NF-κB binding and inhibit DNA looping of the enhancer to the *TNFAIP3* promoter, effectively suppressing *TNFAIP3* expression [57].

3D genome analysis is also a powerful method for identifying unsuspected candidate genes whose expression could be altered by risk variants in enhancers. For example, an enhancer harboring GWAS risk variants for rheumatoid arthritis was identified between oligodendrocyte transcription factor 3 (*OLIG3*) and *TNFAIP3*. In contrast to the well characterized role of *TNFAIP3* in regulating inflammatory signaling pathways, *OLIG3* is an important regulator of neuronal development and has no established role in the immune system [58], suggesting that *TNFAIP3* was the likely target of this enhancer. To test this hypothesis, Capture Hi-C studies in both B and T cells from patients with rheumatoid arthritis were performed. Interestingly, these studies revealed that chromatin loops formed not only between the enhancer and the downstream promoter of *TNFAIP3*, but also with the promoters of interleukin 20 receptor subunit alpha (*IL20RA*) and interferon gamma receptor 1 (*IFNGR1*) located 180 kb upstream [59]. Functional follow-up studies using 3C confirmed that the presence of the risk allele of SNP, rs6927172, in the enhancer resulted in increased looping to the promoter and a concomitant increase in *IL20RA* gene expression [59]. Importantly, no long-range interactions were observed between the enhancer and the *OLIG3* promoter, effectively eliminating this gene from further consideration. Together, these studies demonstrate the utility of targeted 3D genome applications to test and refine hypotheses regarding loop formation with enhancers harboring GWAS risk variants and their impact on gene expression.

Variants that disrupt anchor protein motifs, such as CTCF motifs that define TAD and insulated neighborhood boundaries, also have the potential to disrupt gene expression regulation by permitting aberrant boundary formation and allowing unrestricted enhancer activation of genes normally excluded from the neighborhood (Fig. 4c, d). An example of this occurs in T-cell acute lymphoblastic leukemia (T-ALL) where a mutation in a CTCF anchor motif disrupts the insulated neighborhood where the T-ALL-associated oncogene, TAL BHLH transcription factor 1 (*TAL1*), resides [60]. Importantly, this insulated neighborhood is devoid of a promoter, effectively inhibiting *TAL1* expression. Disrupting this CTCF boundary allows the promoter of a nearby gene, STIL centriolar assembly protein (*STIL*), to reposition near *TAL1* and activate expression [60]. This is one of many reported mutations in CTCF anchor motifs that have been shown to promote tumorigenesis by modifying looping activities around specific oncogenes [32, 61, 62].

3D genome technologies that typically require tens of millions of input cells have largely limited investigations into the potential implications of disrupting CTCF boundaries in primary immune cells involved in autoimmune disease pathogenesis. However, a recent report using 4C with NGS demonstrated that two asthma risk variants at the chromosome 17q21 locus, rs4065275 and rs12936231, individually altered CTCF binding motifs in CD4+ and CD8+ T cells [63]. In both cases, the 3D regulatory networks at this locus were significantly altered, resulting in increased expression of ORMDL sphingolipid biosynthesis regulator 3 (*ORMDL3*), a gene that facilitates cytokine production in the lung [63]. This study is one of the first to demonstrate that chromatin conformation methodologies can be used in primary cells to show that variants disrupting specific insulator boundaries can significantly alter the expression of genes implicated in disease pathogenesis. As more of these studies emerge in the future, we anticipate this to be a recurring theme, not only for cancer but complex diseases as well.

## Conclusions

Analysis of 3D chromatin topology is an essential component for a complete mechanistic understanding of how genetic variants associated with complex human disease drive disease pathogenesis. As large-scale 3D chromatin technologies improve, we anticipate many of their current shortcomings will dissipate. In particular, we look forward to improvements in analytical methods that would facilitate truly quantitative comparisons of relative loop frequencies and enhancer–promoter interactions between different cell types and conditions. This, combined with reductions in chromatin input requirements such that small numbers of primary cells could be analyzed, would scale the potential of 3D chromatin studies in a manner that is now common for transcriptome studies.

The recently established 4D Nucleome Project supported by the NIH Common Fund [64] is charged with developing a “wiring diagram” for how the “parts list” discovered by the ENCODE and NIH-Roadmap consortiums is connected in 3D space and time to orchestrate proper gene transcription. Importantly, this consortium will work to innovate single-cell applications and establish quantitative analytical and innovative visualization platforms to bring 3D genome information into the mainstream of complex disease genetic analysis. As we have attempted to highlight in this review, this knowledge will be necessary for us to fully translate GWAS information into a more precise mechanistic understanding of how genetic variation influences disease risk. Ultimately, we hope that this will lead to improvements in our ability to predict, diagnose, and treat autoimmune diseases.

## Abbreviations

3C: Chromatin conformation capture
3D: Three-dimensional
CTCF: CCCTC-binding factors
ENCODE: Encyclopedia of DNA Elements
GWAS: Genome-wide association study(ies)
H3K27ac: Acetylation of histone H3 on lysine 27
IDH: Isocitrate dehydrogenase
IFNGR1: Interferon gamma receptor 1
IGF2: Insulin-like growth factor 2
IHEC: International Human Epigenome Consortium
IL20RA: Interleukin 20 receptor subunit alpha
NF-κB: Nuclear factor kappa B
NGS: Next generation sequencing
NIH: National Institutes of Health
OLIG3: Oligodendrocyte transcription factor 3
ORMDL3: ORMDL Sphingolipid biosynthesis regulator 3
PCR: Polymerase chain reaction
PDGFRA: Platelet-derived growth factor receptor alpha
SLE: Systemic lupus erythematosus
SNP: Single nucleotide polymorphism
TAD: Topologically associating domain
TAL1: TAL BHLH Transcription Factor 1
T-ALL: T-cell acute lymphoblastic leukemia
TNFAIP3: Tumor necrosis factor alpha-inducible protein 3
